# Collaboration and partnership in orthopaedics and musculoskeletal research: academia, industry, health services, and patients

**DOI:** 10.1186/1471-2474-16-S1-S12

**Published:** 2015-12-01

**Authors:** Arash Angadji

**Affiliations:** 1 Orthopaedic Research UK, 10A Chandos St, London W1G 9DQ, UK

## 

The main activity of Orthopaedic Research UK (ORUK) is to provide funding to centres of excellence to conduct research in the field of orthopaedics, in order to benefit patients suffering from bone and joint disorders. However, the majority of the grants awarded will not make it to the market as products, surgical techniques or novel rehabilitation approaches. Therefore, in general, grants are made with no expectation of a financial return and very often with no measureable patient benefit. But in the event when the researchers manage to generate intellectual property (IP) and a commercial opportunity, it is expected that the funder should also share the financial benefits in order to reinvest the money back into its pursuit of its mission.

Since 2004, ORUK has invested over £8.2m on 120 projects in 37 institutions, which has thus far generated nil return on investment. Even though so far ORUK has managed to file three patents – in 2009, 2013 and 2014 - the organisation has yet to see the long-term benefits for the stakeholders. ORUK is addressing a perceived lack of efficiency to show impact on patients by implementing a new approach through a specific translation research funding (TRF) programme within its strategy. ORUK is aiming to promote the innovation process to ensure that the money invested in research is given the best opportunity to translate into meaningful outcomes.

